# Genetic structure of *Sclerotinia sclerotiorum* populations from sunflower and cabbage in West Azarbaijan province of Iran

**DOI:** 10.1038/s41598-022-13350-7

**Published:** 2022-06-03

**Authors:** Masoumeh Faraghati, Masoud Abrinbana, Youbert Ghosta

**Affiliations:** Department of Plant Protection, Faculty of Agriculture, Urmia University, PO Box 165, Urmia, Iran

**Keywords:** Fungal genetics, Fungal biology

## Abstract

*Sclerotinia sclerotiorum* is one of the most destructive fungal pathogens infecting a wide array of plant species worldwide. Management of this pathogen relies on the coordinated use of fungicides and resistant host cultivars with other control measures, but the effectiveness of these methods requires knowledge of the genetic variability and structure of the fungal populations. To provide insight into the genetic diversity and structure of this pathogen in West Azarbaijan province of Iran, a total of 136 isolates were collected from symptomatic sunflower and cabbage plants within fields in three regions and analysed using inter-simple sequence repeat (ISSR) markers and intergenic spacer (IGS) region of the rRNA gene sequences. A total of 83 ISSR multilocus genotypes (MLGs) were identified, some of which were shared among at least two regional or host populations but in a low frequency. High genotypic diversity, low levels of clonal fraction, and random association of ISSR loci in a region indicated a low level of clonal reproduction, and possibly a high level of sexually recombining life cycle for the pathogen in the province. Marker analyses revealed that the pathogen was spatially homogeneous among fields, and thus similar control measures, such as the choice of resistant cultivars and fungicides, may effectively manage *S. sclerotiorum* within the region. Four IGS haplotypes (IGS1–IGS4) were detected within populations with IGS3 being the most prevalent haplotype. The low IGS haplotype diversity, the absence of spatial structure, and shared MLGs among populations may suggest a single introduction and subsequent dispersal of *S. sclerotiorum* within West Azarbaijan province.

## Introduction

*Sclerotinia sclerotiorum* is a devastating fungal pathogen causing various diseases such as white mold, stem rot, basal stem rot, and fruit rot in a wide range of plant species worldwide^1^. Yield losses posed by this necrotrophic pathogen vary and can reach up to 100% under favorable conditions and severe infection^2,3^.

Sclerotia are considered the prime components in the epidemiology and disease cycle of *S. sclerotiorum* because they are the main surviving structures and provide asexual and sexual inoculum for infection through myceliogenic and carpogenic germination, respectively. In myceliogenic germination, sclerotia produce hyphae that infect the basal stem and roots of plants, whereas, in carpogenic germination, they produce apothecia and wind-blown ascospores that infect aerial plant parts^1,3^. Both reproduction modes can result in a clonal population structure because *S. sclerotiorum* is a homothallic species, and sexual reproduction leads to progenies that do not segregate in any marker and gene^4,5^. Due to the long-term persistence of sclerotia in soil and multiple infection routes, along with a broad host range and lack of highly resistant cultivars, the pathogen has been proven difficult to manage in the field. Nonetheless, coordinated use of fungicides and partially resistant cultivars with other control measures that reduce the pathogen inoculum and manipulate the environment to create unfavorable conditions for the pathogen development, can assist in managing the disease in the field^1,3^. However, the effectiveness of control measures, particularly chemical control and resistant cultivar employment, requires knowledge of the genetic diversity and structure of the fungal populations^6^.

Genetic structure is the outcome of main evolutionary forces, including reproduction/ mating system, gene flow, random genetic drift, selection, and mutation. A thorough understanding of the role of each determinant on genetic structure can assist in predicting the potential risk of a fungal pathogen and improving control strategies^6^. Genetic structure of *S. sclerotiorum* from different hosts and geographic regions has been studied using mycelial compatibility group (MCG)^7–10^ and various molecular markers such as inter-simple sequence repeat (ISSR)^11^, random amplified polymorphic DNA (RAPD)^12^, restriction fragment length polymorphism (RFLP)^7–10^, sequence-related amplified polymorphism (SRAP) technique^13^, simple sequence repeat (SSR)^14–20^, and universal rice primers (URP)^21^. Results of several population genetic analyses have been consistent with homothallic mating and sclerotia production of *S. sclerotiorum* and found a clonal genetic structure with low genotypic diversity, linkage disequilibrium at marker loci, and dominance of one or a few multilocus genotypes (MLGs) over time and space^8–10,22,23^. However, a number of studies have found high genotypic diversity within populations and random association of marker alleles and concluded that recombination (outcrossing) infrequently occurs in the life cycle of *S. sclerotiorum* and contributes to the population structure of the pathogen in the studied regions^14–17,21,24–28^.

West Azarbaijan, located in northwest Iran, is one of the leading agriculture production provinces of the country. This province is ranked first for the cultivation of oilseed and confectionary varieties of sunflower nationwide. Vegetables are also important crops that are grown in various regions of the province, and among which, cabbage is mainly cultivated in Urmia (capital of West Azarbaijan). *Sclerotinia sclerotiorum* is a common pathogen of these crops causing substantial yield losses in the region. Hence, a number of studies have been conducted to understand the MCG and aggressiveness diversity of isolates from sunflower^29,30^ and cabbage^31^ in the province. The genetic diversity of *S. sclerotiorum* isolates from four northern provinces of Iran have been studied using molecular markers^27,32^, but no information is available on genetic diversity and structure of the pathogen populations in West Azarbaijan or other regions of the country. Therefore, this study aimed to determine the genetic variation of *S. sclerotiorum* in sunflower and cabbage fields located in various regions of West Azarbaijan province. The specific objectives were: (a) to determine inter- and intrapopulation diversity using ISSR markers, (b) to infer the relative role of clonal reproduction and recombination in population biology of the pathogen, and (c) to identify haplotypes and to estimate diversity based on intergenic spacer (IGS) region of the rRNA gene sequences.

## Results

### ISSR markers

The seven ISSR primers amplified 65 scorable and reproducible loci in 136 isolates, of which 55 (84.62%) were polymorphic across the dataset (Table 1). Genotype accumulation curve revealed that all MLGs could be discriminated by 39 loci (Supplementary Fig. S1) and, thus, 55 polymorphic loci were highly informative for the analyses.

**Table 1:** Diversity indices and index of association calculated for geographic and host populations of *Sclerotinia sclerotiorum*.

Population	n^a^	B^b^	P^c^ (%)	MLG^d^	eMLG^e^ ± SE	*H*′^f^	*G*′^g^	CF^h^	*H*_exp_^i^	*I*_*A*_^j^	$\overline{r}_{d}$^k^
**Geographic population**											
Urmia	95	65	81.54	59	12.7 ± 1.04	1.52	2.94	0.38	0.22	0.98*	0.020*
Salmas	27	64	66.15	22	12.7 ± 0.87	1.20	1.55	0.19	0.22	0.39	0.009
Khoy	14	64	44.62	14	14.0 ± 0.0	1.00	1.00	0.00	0.17	0.96*	0.035*
Total	136	65	84.62	84	13.1 ± 0.91	1.64	4.13	0.38	0.21	0.92*	0.018*
**Host population**											
Sunflower	93	65	76.92	65	35.8 ± 1.93	1.13	1.40	0.30	0.20	0.59*	0.013*
Cabbage	43	65	76.92	27	27.0 ± 0.0	0.92	0.57	0.37	0.24	1.18*	0.025*
Total	136	65	84.62	84	35.0 ± 2.17	1.19	1.55	0.38	0.21	1.02*	0.018*

^a^Number of isolates.

^b^Number of polymorphic bands.

^c^Percentage of polymorphic bands.

^d^Number of multilocus genotypes.

^e^Expected number of multilocus genotypes.

^f^Corrected Shannon–Weaver index of MLG diversity [*H*/ ln (eMLG)].

^g^Corrected Stoddart and Taylor index of MLG diversity (*G*/ eMLG).

^h^Clonal fraction.

^i^Nei's unbiased gene diversity.

^j^Index of association.

^k^Standardized index of association.

*Significance at *p* < 0.01.

### Genetic diversity and population structure based on ISSR markers

The number of ISSR loci amplified in geographic populations did not differ considerably and ranged from 64 for isolates from Khoy and Salmas to 65 for isolates from Urmia (Table 1). However, the percentage of polymorphic DNA bands varied substantially among the three populations and ranged from 44.62% in Khoy to 81.54% in Urmia. The primers yielded the same number of loci (65) with a similar level of polymorphism (76.92%) in the isolates from the two hosts (Table 1).

**Figure 1:**
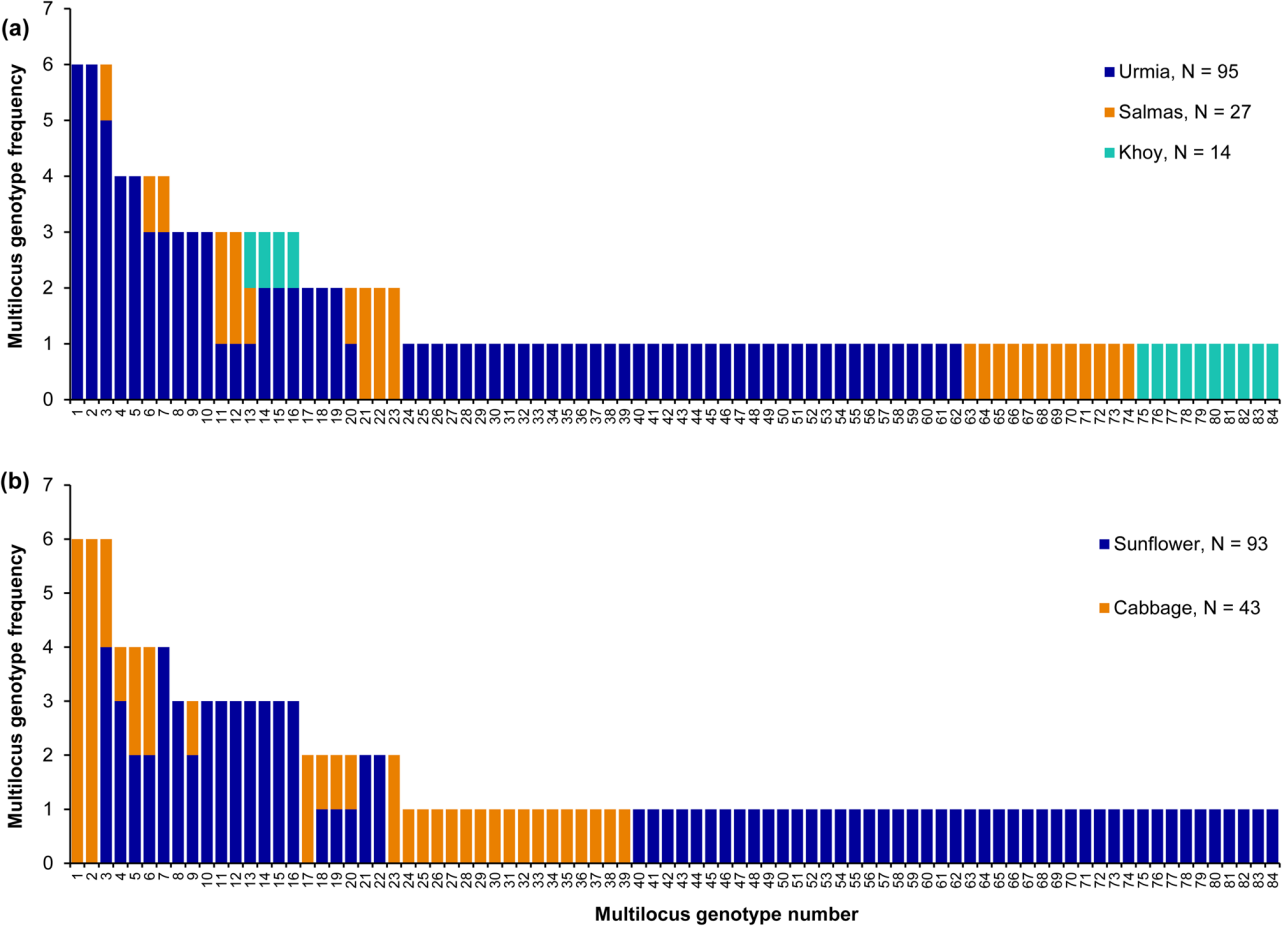
Frequency of ISSR multilocus genotypes (MLGs) for 136 isolates of *Sclerotinia sclerotiorum* from geographic (**a**) and host (**b**) populations in West Azarbaijan province of Iran.

Within-population diversity indices were estimated for fungal populations from geographic regions and host crops separately (Table 1). A total of 84 MLGs were identified within 136 *S. sclerotiorum* isolates (Table 2, Fig. 1). Twenty-three MLGs shared by two to six isolates and, thus, they were clonal (Fig. 1). Clones of 13 MLGs were found within the same region, but isolates of nine MLGs were shared between two geographic populations, and clonal isolates of one MLG (MLG13) were detected in the three studied regions (Fig. 1a). Clones of 15 MLGs were found exclusively in sunflower or cabbage fields, whereas eight MLGs represented by the isolates from the two sampled hosts (Fig. 1b).

**Table 2:** Population differentiation (Ф_PT_, above diagonal) for pairwise comparisons among geographic populations of *Sclerotinia sclerotiorum* and overall value for all populations.

	Urmia	Salmas	Khoy
Urmia	–	0.012 ns	0.00 ns
Salmas	–		0.00 ns
Khoy	–	–	–
Ф_PT_ (all populations)	0.004		

ns, non significant.

The number of observed MLGs in geographic populations varied from 14 in Khoy to 59 in Urmia (Table 1). The highest genotype richness (eMLG = 14.0) was observed in Khoy population, while Urmia and Salmas had a lower richness (eMLG = 12.7). The Urmia population showed the highest genotypic diversity based on *G´* (2.59) and *H´* (1.52), while Khoy had the lowest genotypic diversity (*G´* = 1.00 and *H´* = 1.00). Clonal fraction also differed across geographic populations and varied from 0.00 in Khoy to 0.38 in Urmia. The mean gene diversity (*H*_exp_) of the studied populations was 0.21. The highest *H*_exp_ (0.21) was estimated in Urmia and Salmas, while the Khoy had a lower (0.17) gene diversity (Table 1).

The fungal populations sampled from the two host plants also differed in their diversity (Table 1). The pathogen population from sunflower had higher eMLG (35.8), *G´* (1.40) and *H´* (1.13) as well as lower clonal fraction (0.30) compared to the population from cabbage (eMLG = 27.0, *G´* = 0.57, *H´* = 0.92, and clonal fraction = 0.37). In contrast, *H*_exp_ in the cabbage population was found to be higher (0.24) compared to the sunflower population (0.20) (Table 2).

AMOVA analyses of clone-corrected datasets revealed that 0.0% (Ф_PT_ = 0.004, *P* = 0.25; Table 2) and 1% (Ф_PT_ = 0.012, *P* = 0.07) of genetic variation could be attributed to the differences among geographic populations and between the samples collected from the two hosts, respectively. Pairwise comparisons of geographic populations also indicated a nonsignificant genetic differentiation between them (Table 2).

**Figure 2:**
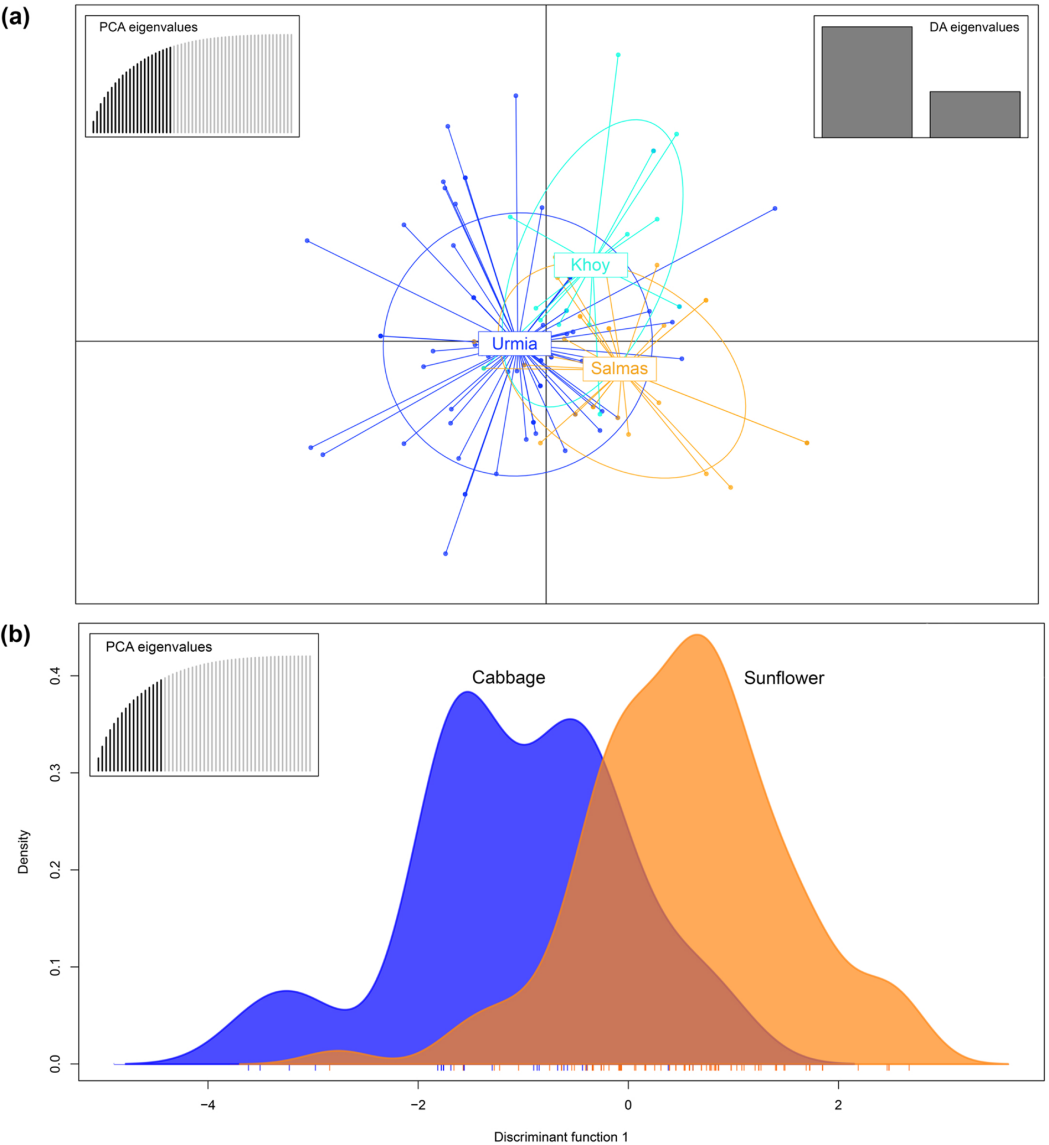
Scatter plot (**a**) and density plot on the first discriminant function (**b**) showing discriminant analysis of principal components (DAPC) for ISSR multilocus genotypes (MLGs) of *Sclerotinia sclerotiorum* from geographic and host populations, respectively. Dots represent MLGs.

The nonsignificant genetic differentiation revealed by AMOVA analyses was also confirmed by DAPC analyses using predefined geographic (Fig. 2a) and host (Fig. 2b) populations. The analyses indicated that MLGs of the geographic populations were aggregated in DAPC scatter plot (Fig. 2a), and the density plots of the host populations were mostly overlapped (Fig. 2b).

Bayesian clustering using STRUCTURE software identified three clusters based on the magnitude of *ΔK* (Supplementary Fig. S2), but estimates of ln *K* inferred four genetic clusters within populations (Supplementary Fig. S3). The DAPC resulted in a different estimate of optimal *K*, and the optimal number of clusters revealed by the lowest BIC score was 5 (Supplementary Fig. S4). Assignment of 84 MLGs to the three, four, and five inferred clusters indicated that membership coefficients of 10 (11.90%), three (3.57%), and four (4.76%) MLGs, respectively, were more than 0.8 (0.81–0.95) and could be assigned to the inferred genetic groups whereas the remaining MLG were admixed (Supplementary Fig. S5).

**Figure 3:**
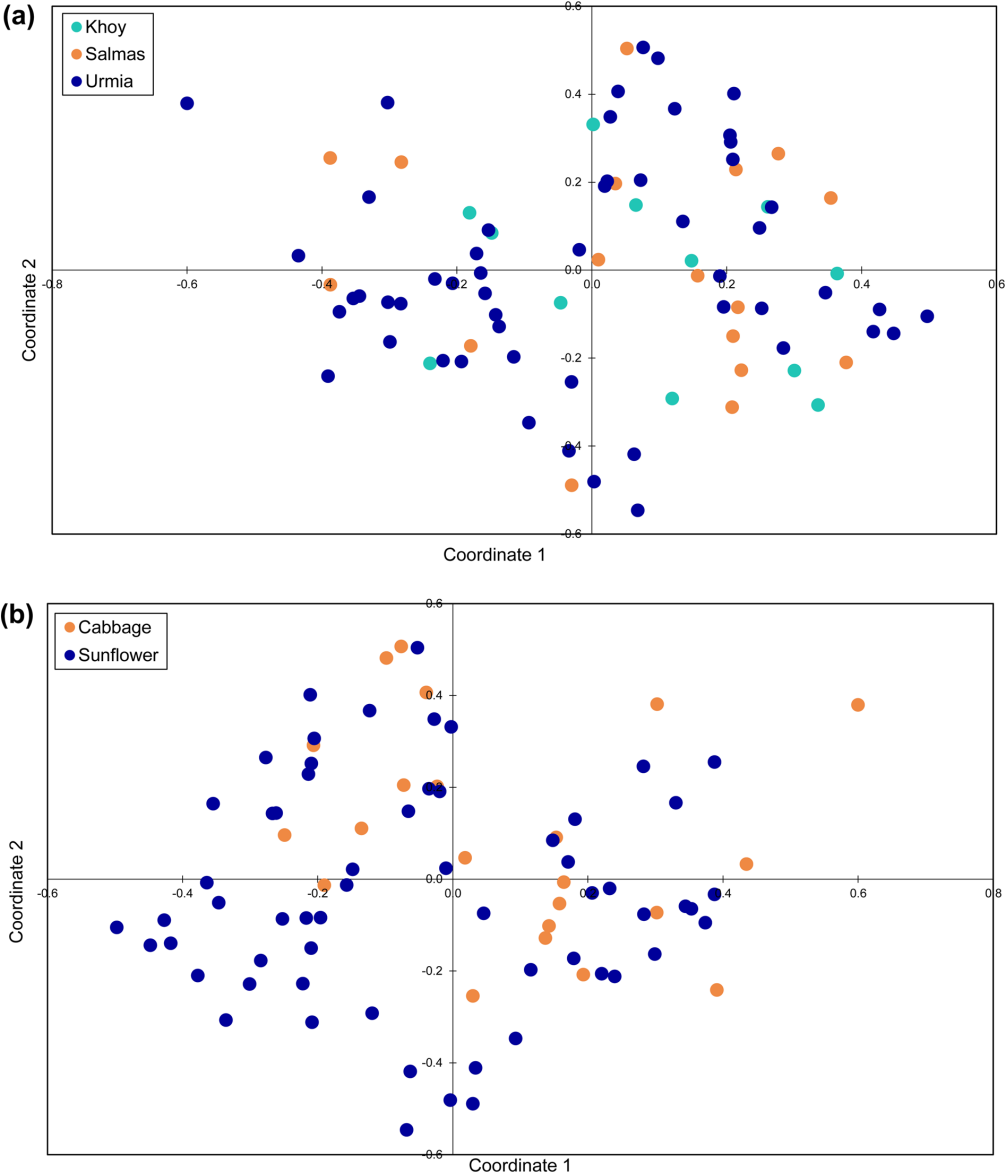
Principal coordinate analysis for ISSR multilocus genotypes (MLGs) of *Sclerotinia sclerotiorum* from geographic (**a**) and host (**b**) populations using Jaccard similarity coefficient.

**Figure 4:**
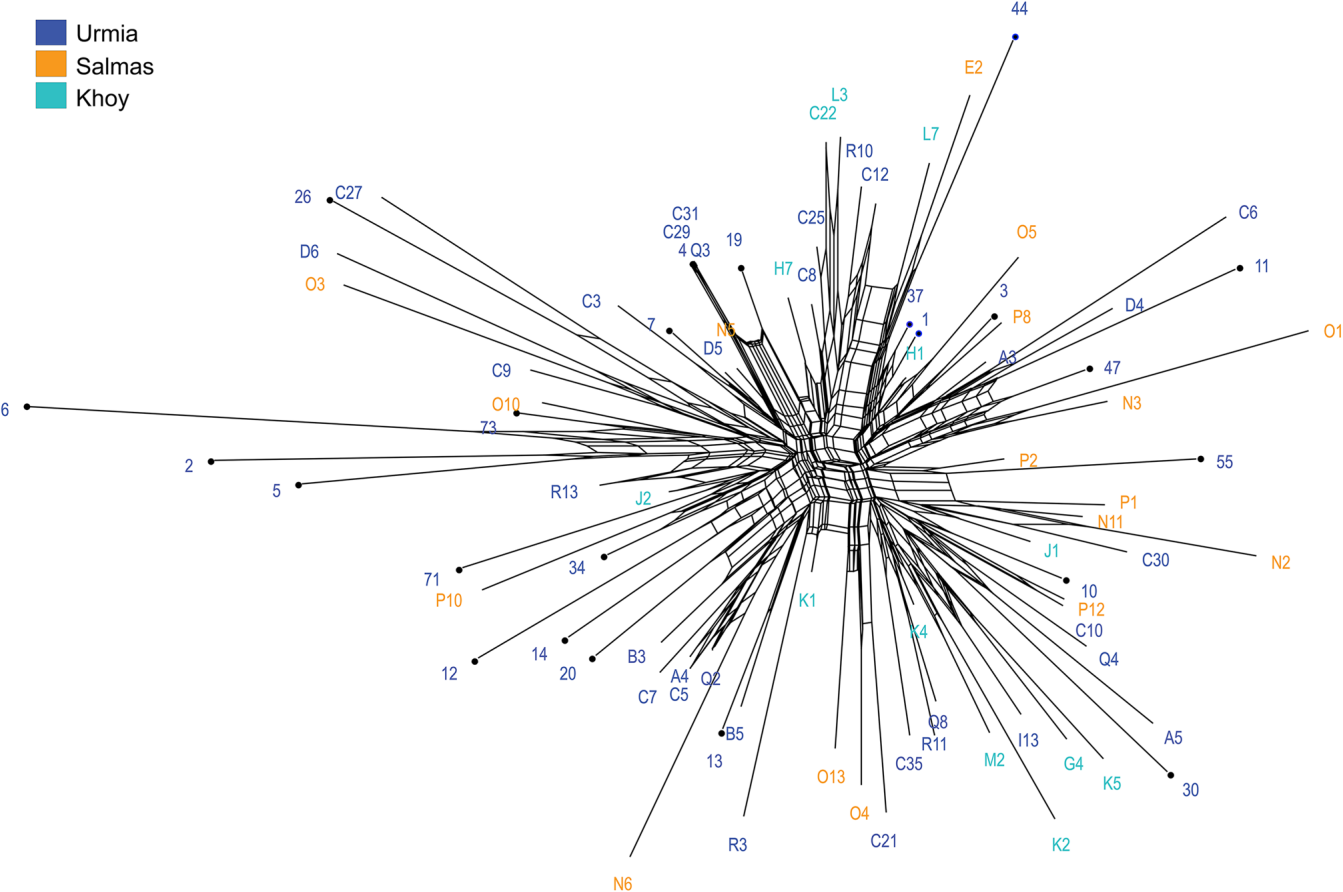
Neighbor-Net graph for 84 ISSR multilocus genotypes (MLGs) of *Sclerotinia sclerotiorum* from sunflower and cabbage fields in three regions (Urmia, Salmas and Khoy) in West Azarbaijan province of Iran. The isolates from cabbage fields are indicated by black circles at the nodes. Reticulations reveal possible recombination events.

In PCoA analyses, MLGs from geographic (Fig. 3a) and host (Fig. 3b) populations were randomly distributed, and no grouping of the individuals was observed in the plots. The result of Neighbor-Net (Fig. 4) was in agreement with the results of PCoA. The star-like topology of the network revealed close genetic relationships of the MLGs and rejected the clustering of MLGs into distinct genetic groups. In Neighbor-Net, a number of reticulations were also observed, indicating the occurrence of recombination between the MLGs (Fig. 4).

Tests for recombination in clone-corrected geographic and host populations indicated that ISSR loci in Salmas were randomly associated whereas, in other populations, indices of multilocus linkage disequilibrium (*I*_*A*_ and $\overline{r}_{d}$) were significantly (*p* < 0.01) higher than zero (Table 2 and Supplementary Figs. S6 and S7).

### IGS sequence analysis and haplotype diversity

**Figure 5:**
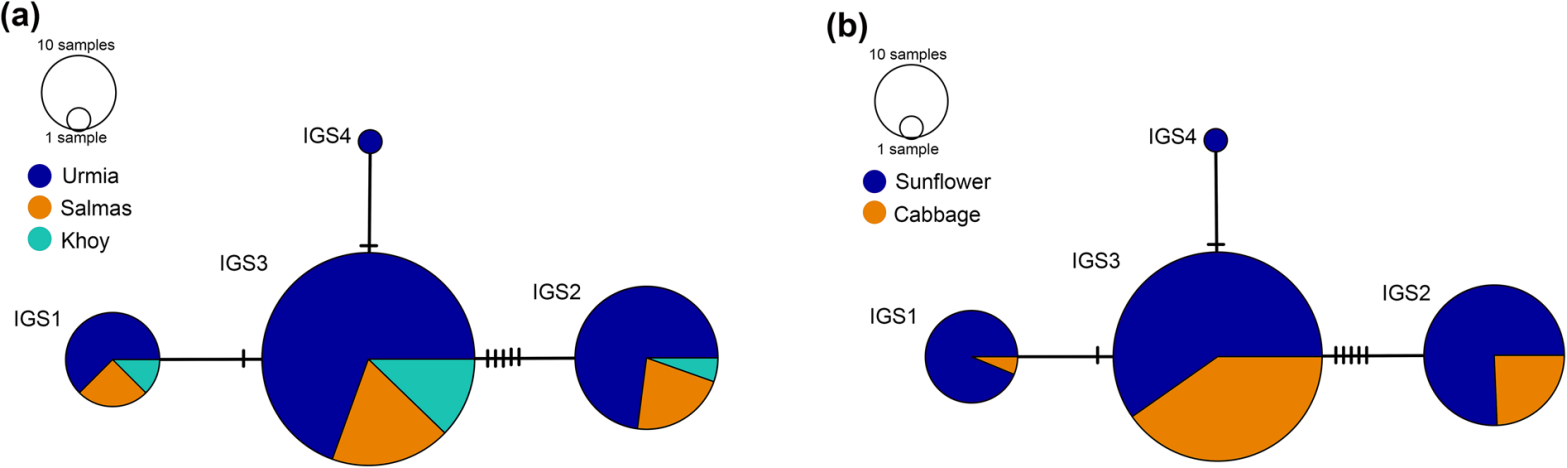
Median-joining networks displaying the relationships between IGS haplotypes of *Sclerotinia sclerotiorum* from West Azarbaijan province of Iran and their distribution in geographic (**a**) and host (**b**) populations. Each circle indicates a single haplotype and the size of circle corresponds to the frequency of that haplotype. Each hatch mark on the lines joining haplotypes, represents one nucleotide substitution.

Analysis of IGS sequences revealed a total of four haplotypes within 136 isolates. Comparison of our sequences with those from previously known haplotypes indicated that they belonged to IGS1, IGS2, IGS3, and IGS4 (Table 3 and Fig. 5). Among the identified haplotypes, IGS3, which contained 82 isolates, was the most common across all regions on both host crops. The two haplotypes, IGS2 (37 isolates) and IGS1 (16 isolates), were also detected in all populations. Among the studied isolates, only one isolate, which was collected from a sunflower field in Urmia, was belonging to IGS4 (Table 3 and Fig. 5). Overall IGS haplotype diversity was 0.553, but the value of this index in geographic populations differed from 0.484 in Khoy to 0.649 in Urmia. The IGS haplotype diversity (0.649) of the sunflower population was higher compared to the diversity (0.375) observed in the fungal population from cabbage (Table 3).

**Table 3:** Frequency and diversity of IGS haplotypes of *Sclerotinia sclerotiorum* isolates in geographic and host populations.

Population	IGS haplotype				No. isolates	No. haplotypes	Haplotype diversity
	IGS1	IGS2	IGS3	IGS4			
**Geographic population**							
Urmia	10	27	57	1	95	4	0.649
Salmas	4	8	15	0	27	3	0.604
Khoy	2	2	10	0	14	3	0.484
**Host population**							
Sunflower	15	28	49	1	93	4	0.649
Cabbage	1	9	33	0	43	3	0.375
Total	16	37	82	1	136	4	0.553

## Discussion

In this study, the diversity of *S. sclerotiorum* populations in West Azarbaijan province of Iran was investigated by using a total of 136 fungal isolates from two host crops and three geographic regions, and ISSR markers and IGS sequence data. Analyses of ISSR data revealed low clonal fraction, high genotypic diversity, and genetic homogeneity across geographic and host populations. Based on IGS data, low haplotype diversity was observed within the *S. sclerotiorum* populations suggesting that the pathogen populations in the province may be derived from a founding population.

The populations of *S. sclerotiorum* in West Azarbaijan consisted of 84 ISSR MLGs, and the majority (61 MLGs, 72.62%) had only one isolate, while 23 genotypes (27.38%) had two or more isolates indicating clonal individuals. Clones of three MLGs were sampled in a relatively higher frequency than the others, but only MLG3 was widely distributed and was present in isolates from the two hosts in the two regions. In addition, estimation of association in ISSR loci indicated significant gametic disequilibrium in the host populations and two geographic populations. These findings were consistent with previous reports on *S. sclerotiorum* from various hosts and regions^7–10,22,23,33–35^ and revealed the occurrence of clonal reproduction through sclerotia and/or self-fertilization in the pathogen populations in the province.

Despite the clonality in the studied *S. sclerotiorum* populations, the frequencies of the most prevalent MLGs were not high, and the overall proportion of distinct MLGs was 0.62. Molecular marker studies of *S. sclerotiorum* populations have revealed MLG proportions of 0.12 for various crops in New York^36^, 0.29 for canola in northern Iran^27^, 0.45 for dry bean in the USA, France, Australia, and Mexico^37^, 0.51 for common bean in Argentina^21^, 0.58 for rapeseed in China^20^, 0.59 for various crops and meadow buttercup in the UK^16^, 0.59 for common bean in Brazil^18^ and 0.61 for various crops in England, Wales, Scotland, Norway and Australia^17^. Furthermore, in the present study, proportions of MLGs ranged from 0.62 to 1 in geographic populations and were 0.70 and 0.63 in the samples collected from sunflower and cabbage, respectively. The maximum MLG proportion and the absence of clones in Khoy population could be due to the limited number of isolates collected from this region, nevertheless, the ranges of MLG proportions in the populations were generally larger than those found in northern provinces of Iran (0.21–0.45)^27^ and several other regions such as UK (0.24–0.72)^16^ and Australia (0.28–0.68)^28^. The different MLG proportions observed in West Azarbaijan and northern provinces of Iran^27^ may be attributable to the difference in sample sizes, sampling strategies, and spatial scales evaluated in the two studies; the present study investigated three geographic populations within the province, while in the previous study^27^, samples from each of the four provinces were considered as a single population. Altogether, a comparatively high genotypic diversity observed in this study, as well as the low levels of clonal fraction and presence of reticulations in Neighbor-Net, could indicate that *S. sclerotiorum* in West Azarbaijan has not been under strict clonal reproduction and recombination has played a role in the life cycle of the pathogen in the region. However, recombination rates appear to have varied in different populations so that ISSR alleles only in Salmas population were found to be in random association, and the other populations were in gametic disequilibrium.

Parameters of within-population diversity provided some evidence for variation among the *S. sclerotiorum* populations; however, AMOVA analyses indicated the absence of significant variation among the studied populations in West Azarbaijan. The fungal populations from the two host plants showed a very low but nonsignificant level of differentiation in AMOVA and were partly non-overlapped in DAPC. However, the occurrence of shared MLGs between the two populations and a very low inter-population variation (1%) suggest that host identity has not been a barrier to gene flow and MLGs have moved between crops. The observed loose structure might be the result of genetic drift or slight selection pressure exerted by the host plants, but further investigations are needed to precisely unravel processes underlying the very low level of differentiation between the two populations. Although STRUCTURE and DAPC detected various numbers of genetic clusters within populations, the analyses were inconclusive because very few MLGs were assigned to the inferred clusters^38^. It is documented that several factors such as uneven sampling across existing population structure and migration rates between groups can significantly affect the results of admixture methods (STRUCTURE and DAPC) and lead to misspecification of genetic groups. Therefore, it is recommended that the data should additionally be investigated with ordination-based or other methods in order to confirm the results of admixture methods and to determine genetic structuring within populations effectively^39–41^. In the present study, population structure analyses based on PCoA and Neighbor-Net yielded different results compared to the admixture methods, and revealed the absence of genetic structuring in the populations. These findings could indicate that *S. sclerotiorum* isolates likely represent a genetically homogeneous or a very loosely structured population; nevertheless, further study using additional isolates and other molecular markers is suggested to provide a more accurate inference on the population structure of this pathogen in the province.

Analysis of IGS sequences in *S. sclerotiorum* isolates revealed the presence of four haplotypes with various frequencies in West Azarbaijan province. IGS3, IGS2, and IGS1 (82, 37, and 16 isolates, respectively) were the most frequent haplotypes and were widely distributed across most studied regions. Although the information on IGS haplotypes of this pathogen in Iran or other Asian countries is lacking, IGS analysis of *S. sclerotiorum* isolates in other continents indicated that IGS3 is common on various host plants in Europe, North America, and Australia and is the most frequent haplotype in England, New Zealand and the USA^16,17^. The two haplotypes, IGS1 and IGS2, have been found within the fungal populations in all studied countries except Australia and were the next most abundant haplotypes (after IGS3) in England^17^. IGS4 was also detected in a sunflower field in Urmia, which has previously been reported only from England^16,17^. Taken together, the results revealed that IGS haplotype composition and frequency in populations of *S. sclerotiorum* in West Azarbaijan is more similar to England populations than the other countries. This may suggest that both populations have originated from the same ancestral population, but confirmation of this hypothesis needs further investigation. Overall IGS haplotype diversity (0.553) in West Azarbaijan was found to be lower compared with those reported for Australia (0.663), England (0.659), and Scotland (0.632)^17^, suggesting that the studied populations likely descended from a founding population.

In conclusion, our results indicated that *S. sclerotiorum* in West Azarbaijan province has a low IGS haplotype diversity. Furthermore, ISSR marker data revealed the absence of genetic structure based on geographic or host origins, high levels of admixture, and sharing some MLGs among the populations. These findings may suggest the introduction and subsequent dispersal of the pathogen within the province. Clonal reproduction was shown to occur in the region, but the presence of high genotypic diversity, low clonal fraction, and random association of ISSR alleles in a geographic population provided some evidence for the occurrence of recombination in various regions. *Sclerotinia sclerotiorum* is thus able to create new genotypes through recombination and to propagate well-adapted genotypes through clonal reproduction, which can facilitate the evolution of the pathogen against fungicides and resistant cultivars in the province. Since the pathogen is spatially homogeneous among fields within the province, similar control measures, including the choice of resistant cultivars and fungicides, may effectively manage *S. sclerotiorum* across the region.

## Methods

### *Sclerotinia sclerotiorum* isolates

A total of 136 *S. sclerotiorum* isolates obtained from sclerotia collected from infected sunflower and cabbage plants in the fields located in West Azarbaijan, Iran, were used in this study (Supplementary Fig. S8). Ninety-three pure isolates have already been obtained from sunflower fields in Urmia, Khoy, and Salmas^42^. Furthermore, 43 isolates were randomly collected from naturally infected cabbage plants showing head rot symptoms in the fields located in Urmia from July to August 2018. Isolations were performed from sclerotia, and the isolates were purified by transferring hyphal tips grown from single sclerotia onto potato dextrose agar (PDA), as described before^42^. Only one isolate was obtained from each of the sampled plants and used for subsequent studies. The isolates were grouped into three geographic (Urmia, Khoy, and Salmas; Supplementary Fig. S8) and two hosts (sunflower and cabbage) populations and they were analysed separately.

### DNA extraction and ISSR genotyping

Genomic DNA of all *S. sclerotiorum* isolates was extracted from mycelia grown in potato dextrose broth (PDB) using the method of Dellaporta et al.^43^. A total of 40 ISSR primers were initially screened for their ability to amplify discernible and informative band patterns in a subset of isolates and, finally, seven primers (Table S1) that generated polymorphic and reproducible bands were used for genotyping of all isolates. PCR reactions were performed in a total volume of 20 µl and consisted of 10–15 ng genomic DNA, 1X Taq DNA Polymerase 2X Master mix Red (Ampliqon, Denmark), and 0.625 µM primer. Amplifications were carried out on a Corbett CG1-96 Palm-Cycler™ Thermal Cycler (Corbett Life Science, Australia). The thermocycler programs for amplifications were 94 °C for 5 min, followed by 40 cycles of 94 °C for 1 min, annealing at primer-specific temperature (Supplementary Table S1) for 1 min, and 72 °C for 2 min, with a final extension at 72 °C for 10 min. The amplified DNA products were separated on 1.3% agarose gels containing Nucleic Acid Gel Stain (SMOBIO, Taiwan) in 1X TBE buffer. The consistency of ISSR profiles was assessed by replicating DNA extractions, PCR amplifications, and electrophoreses for ten isolates.

### Analysis of ISSR data

The presence or absence of amplified fragments of a particular size was scored as 1 and 0, respectively, and the resulting binary matrix was subjected to statistical analyses. The haplotype or multilocus genotype (MLG) was constructed for each isolate by combining the data from seven ISSR primers, and the isolates with identical banding patterns were assigned to the same MLG (clone). To determine the minimum number of ISSR loci required in discriminating MLGs and accuracy of amplified loci in representing genetic diversity in the studied populations, a genotype accumulation curve was constructed by 1000 times randomly sampling of loci in the package *poppr* v. 2.8.7^44^ for R v. 3.6.3^45^.

The number of amplified DNA fragments and percentage of polymorphic bands were calculated in GenAlEx v. 6.5^46^. Nei's unbiased gene diversity (*H*_exp_)^47^ was estimated in the package *poppr* v. 2.8.7^44^.

To assess the diversity within populations, the number of MLGs and other parameters of MLG diversity were calculated for the full dataset and each of the geographic and host populations separately. As the populations had unequal sample sizes, their genotypic richness was compared by calculating the expected number of MLGs (eMLGs) using the rarefaction method corrected for the smallest sample sizes of geographic (*n* = 14) and host (*n* = 43) populations^48^ in the package *poppr* v. 2.8.7^44^. Genotypic diversity indices *G*^49^ and *H* (Shannon and Weaver index)^48^ were also estimated in *poppr* v. 2.8.7^44^, and were further divided by *g*_max_ (the number of eMLG) and ln(*g*_max_), respectively, so that both indices could be compared for populations with different sample sizes^48^. Clonal fraction for populations was calculated using the formula: 1- (number of MLG/ number of isolates)^50^. All further analyses were conducted on clone-corrected dataset in which only one representative isolate per MLG was retained in the populations.

Genetic differentiation among populations was estimated by calculating the Ф-statistic through analysis of molecular variance (AMOVA)^51^. The Ф-statistic values were calculated in GenAlEx v. 6.5^46^, and their significances were assessed based on 999 permutations of haplotypes.

To examine possible population structure and admixture of isolates, we used the software STRUCTURE v. 2.3^52^ that implements a model-based Bayesian clustering approach to identify the optimal number of genetic clusters (*K*) and assign individuals to the clusters based on genetic similarity. The data were analysed by running a series of simulations for any given *K* between 1 and 10, assuming an admixture model and correlated allele frequencies. For each *K* value, ten independent simulations were conducted with a run length of 200,000 Markov Chain Monte Carlo (MCMC) iterations after a burn-in of 50,000 iterations. STRUCTURE results were compiled with STRUCTURE HARVESTER^53^, and graphical representations of structural plots were constructed using STRUCTURE PLOT v. 2.0^54^. To examine further the population structure, discriminant analysis of principal components (DAPC)^55^ was run in *adegenet* v. 2.1.0 package^56^ for R v. 3.6.3. DAPC is a hypothesis-free method that combines principal component analysis (PCA) and discriminant analysis (DA) to maximize variability between clusters and minimize variability within clusters, without presumptions of Hardy–Weinberg equilibrium or linkage disequilibrium. The optimal number of clusters was identified by find.clusters function, which implements successive *K*-means clustering with increasing values of *K*. The most probable number of clusters was inferred by identifying the lowest Bayesian Information Criterion (BIC)^55^. The optimal number of principal components (PCs) retained in the analyses was determined using xval.dapc function in *adegenet* v. 2.1.0^56^. The genetic clustering inferred by Bayesian clustering and DAPC methods were compared to the results obtained from principal coordinate analysis (PCoA) in GenAlEx v. 6.5^46^ and Neighbor-Net in SplitsTree v. 4^57^. Both later analyses were carried out using Jaccard’s coefficient of similarity.

To test for possible recombination in the populations, index of association (*I*_*A*_) and unbiased estimate of multilocus linkage disequilibrium ($\overline{r}_{d}$)^58^ indices were calculated in the package *poppr* v. 2.8.7^44^, and their deviation from the null hypothesis of linkage equilibrium was assessed by 1000 permutations per population dataset.

### IGS amplification and sequencing

A portion of the IGS region in *S. sclerotiorum* isolates was amplified using primer pair IGS2F/IGS2R^16^. PCR reactions were performed in a total volume of 25 µl and consisted of 10–15 ng genomic DNA, 1X Taq DNA Polymerase 2X Master mix Red (Ampliqon, Denmark), and 0.25 µM each of the forward and reverse primers. Amplifications were carried out on a Corbett CG1-96 Palm-Cycler™ Thermal Cycler (Corbett Life Science, Australia). The thermocycler programs for amplifications were 94 °C for 4 min, followed by 40 cycles of 94 °C for 30 S, 57 °C for 30 S, and 72 °C for 1 min, with a final extension at 72 °C for 10 min. PCR products were purified and sequenced by Beijing Genomics Institute (China).

### Analysis of IGS sequence data

The IGS sequences were checked and edited with BioEdit v. 7.2.0^59^. The sequences of 26 IGS haplotypes^16,17^ were retrieved from GenBank and aligned with sequences generated in this study using MAFFT v. 7.450^60^. IGS haplotypes of our isolates were determined in DnaSP v. 5^61^, and haplotype diversity of populations were calculated using the same software. To visualize the relationships among haplotypes from populations, median-joining networks were reconstructed in PopART v. 1.7^62^.

### Experimental research statement

Experimental research, including the collection of plant material, complied with relevant institutional, national, and international guidelines and legislation.

## Supplementary Information

Supplementary Information.

## Data Availability

The datasets used and/or analysed during the current study are available from the corresponding author on reasonable request.
