# Supplementary material for: Genetic structure of Sclerotinia sclerotiorum populations from sunflower and cabbage in West Azarbaijan province of Iran
Source: Sci Rep. 2022 Jun 3;12:9263. doi: 10.1038/s41598-022-13350-7 (PMC9166751; doi:10.1038/s41598-022-13350-7)
Supplement: Supplementary file 1 — Supplementary Information. [file 41598_2022_13350_MOESM1_ESM.docx]

**Genetic structure of *Sclerotinia sclerotiorum* populations from sunflower and cabbage in West Azarbaijan province of Iran**

Masoumeh Faraghati^1^, Masoud Abrinbana^1*^ & Youbert Ghosta^1^

^1^Department of Plant Protection, Faculty of Agriculture, Urmia University, PO Box 165, Urmia, Iran

^*^email: m.abrinbana@urmia.ac.ir

**Supplementary Table S1.** The name, sequence and annealing temperature of seven ISSR primes used for genotyping *Sclerotinia sclerotiorum* isolates

| **ISSR primer** | **Sequence** | **Annealing temperature (ºC)** | **Reference** |
| --- | --- | --- | --- |
| ISSR 5 | YHY(GT)_7_ | 54 | Zeynali Bari et al. 2021 |
| ISSR 16 | (AG)_8_R | 49 | This study |
| ISSR 17 | (AAG)_8_ | 50 | Groppe et al. 1995 |
| P11 | (AG)_8_Y | 49 | This study |
| P15 | (GACA)_4_ | 48 | Shehata et al. 2008 |
| LMB-A | (GACA)_4_W | 48 | This study |
| UBC 841 | (GA)_8_YC | 50 | Chokheli et al. 2016 |

**References**

Zeynali Bari, R., Abrinbana, M. & Ghosta, Y. Genetic variation, vegetative compatibility, and aggressiveness diversity of Diplodia bulgarica isolates from apple orchards in West Azarbaijan province of Iran. *Plant Pathol* **70**, 1326–1341 (2021).

Groppe, K., Sanders, I., Wiemken, A. & Boller, T. A microsatellite marker for studying the ecology and diversity of fungal endophytes (*Epichloë* spp.) in grasses. *App. Environ. Microbiol.* **61,** 3943–3949 (1995).

Shehata, A. S. *et al.* Single-step PCR using (GACA)4 primer: utility for rapid identification of dermatophyte species and strains. *J. Clin. Microbiol.* **46,** 2641–2645 (2008).

Chokheli, V., *et al.* Preliminary comparative analysis of phenological varieties of *Quercus robur* by ISSR-markers. *J. Bot.* **2016,** 1–7 (2016).


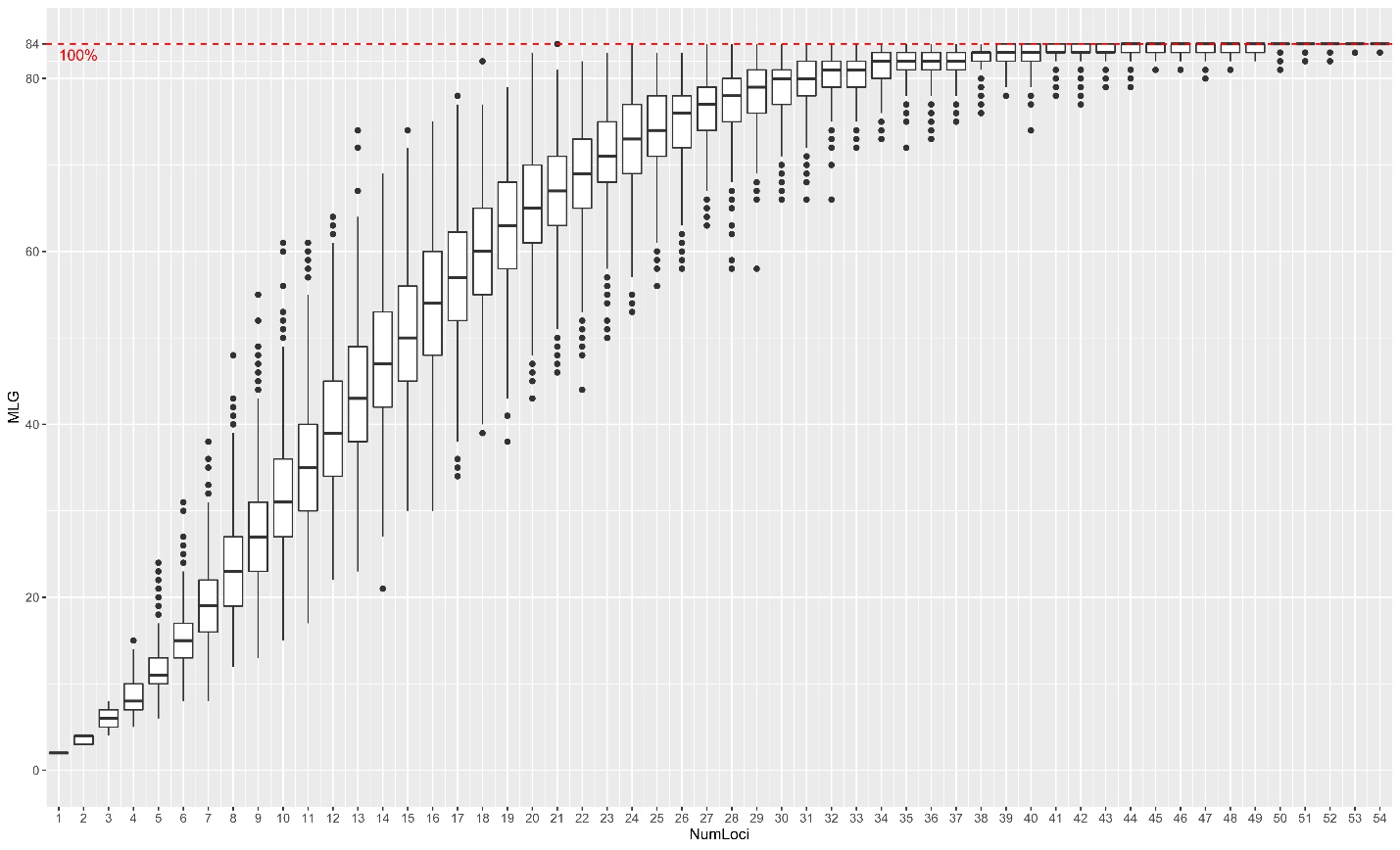


**Supplementary Figure S1.** Genotype accumulation curve for 136 isolates of *Sclerotinia sclerotiorum* generated by 1000 sampling of ISSR loci.


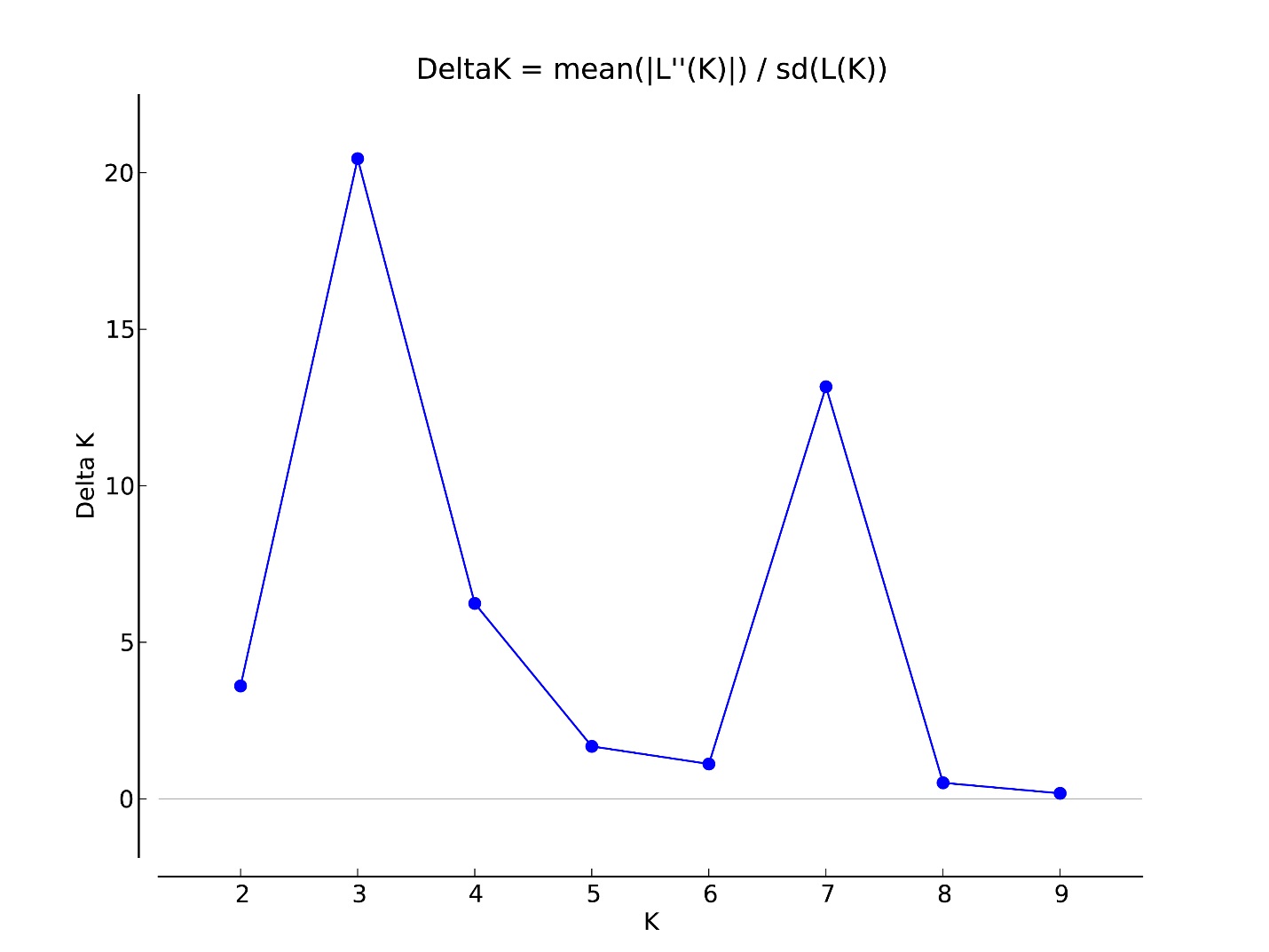


**Supplementary Figure S2.** Result of STRUCTURE HARVESTER showing *ΔK* for each level of *K* inferred from ISSR data using STRUCTURE.


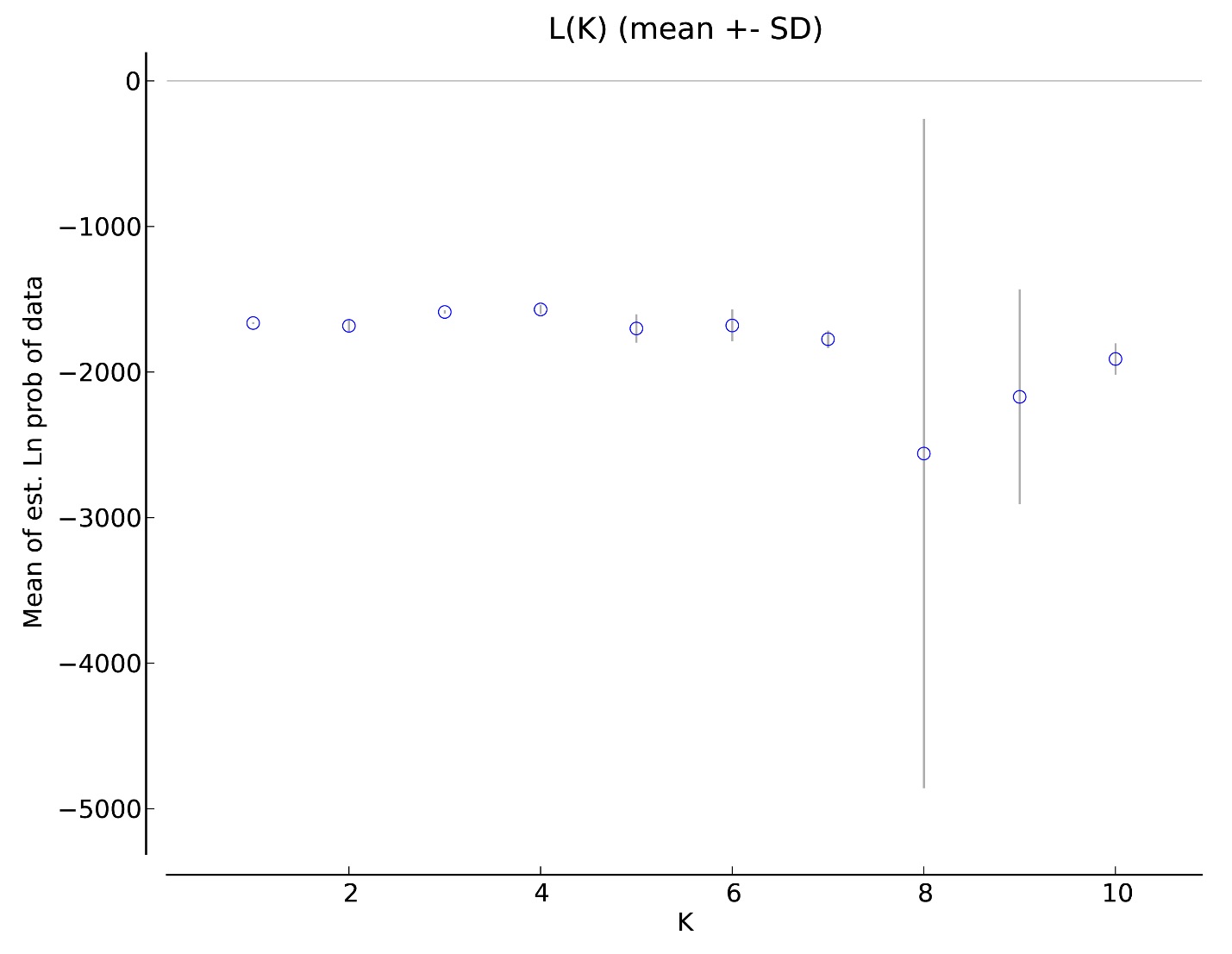


**Supplementary Figure S3.** Result of STRUCTURE HARVESTER showing ln *K* for each level of *K* inferred from ISSR data using STRUCTURE.


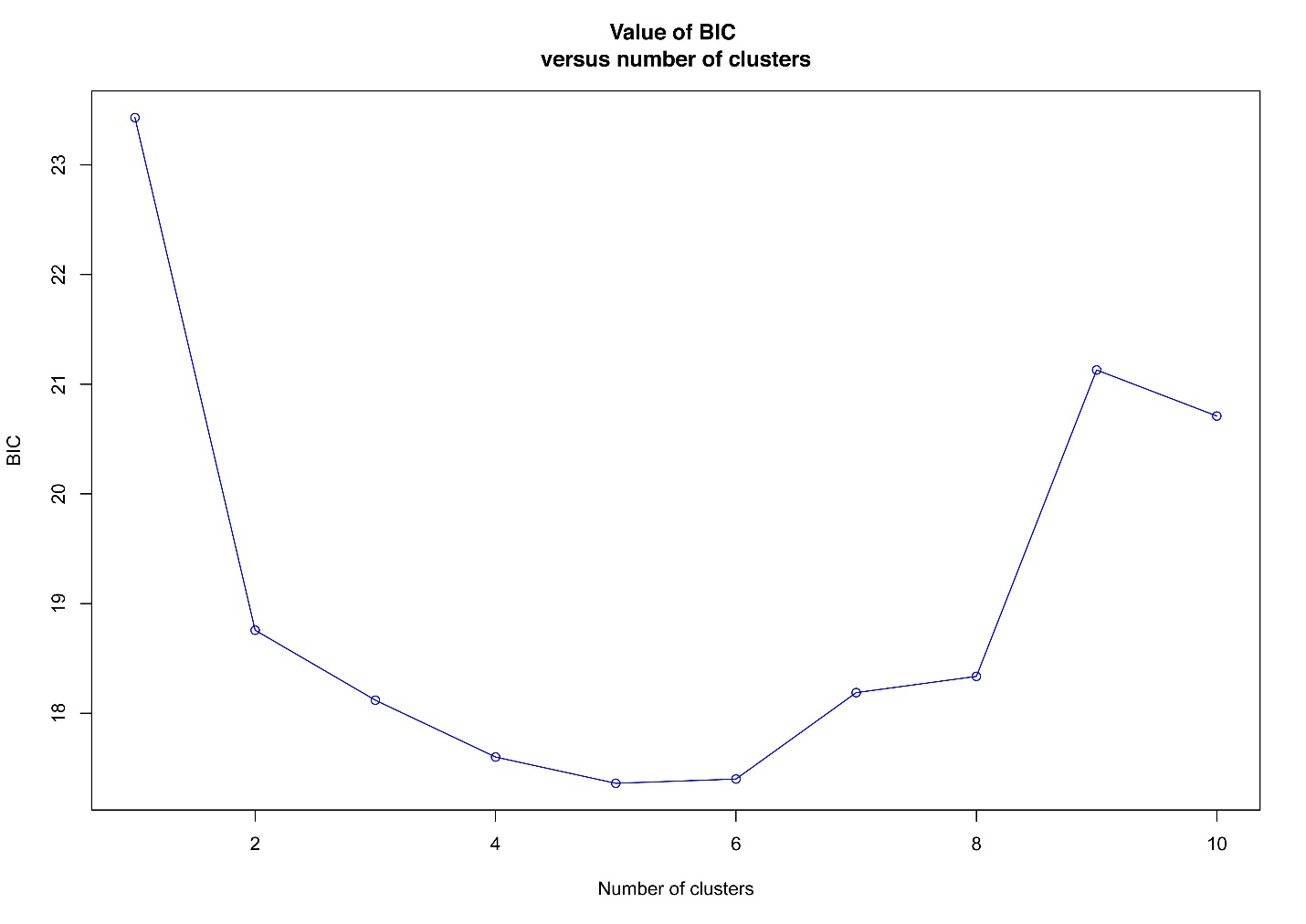


**Supplementary Figure S4.** Bayesian Information Criterion (BIC) values for the genetic clusters calculated with *K*-means clustering using ISSR data for *Sclerotinia sclerotiorum* genotypes from West Azarbaijan province of Iran.


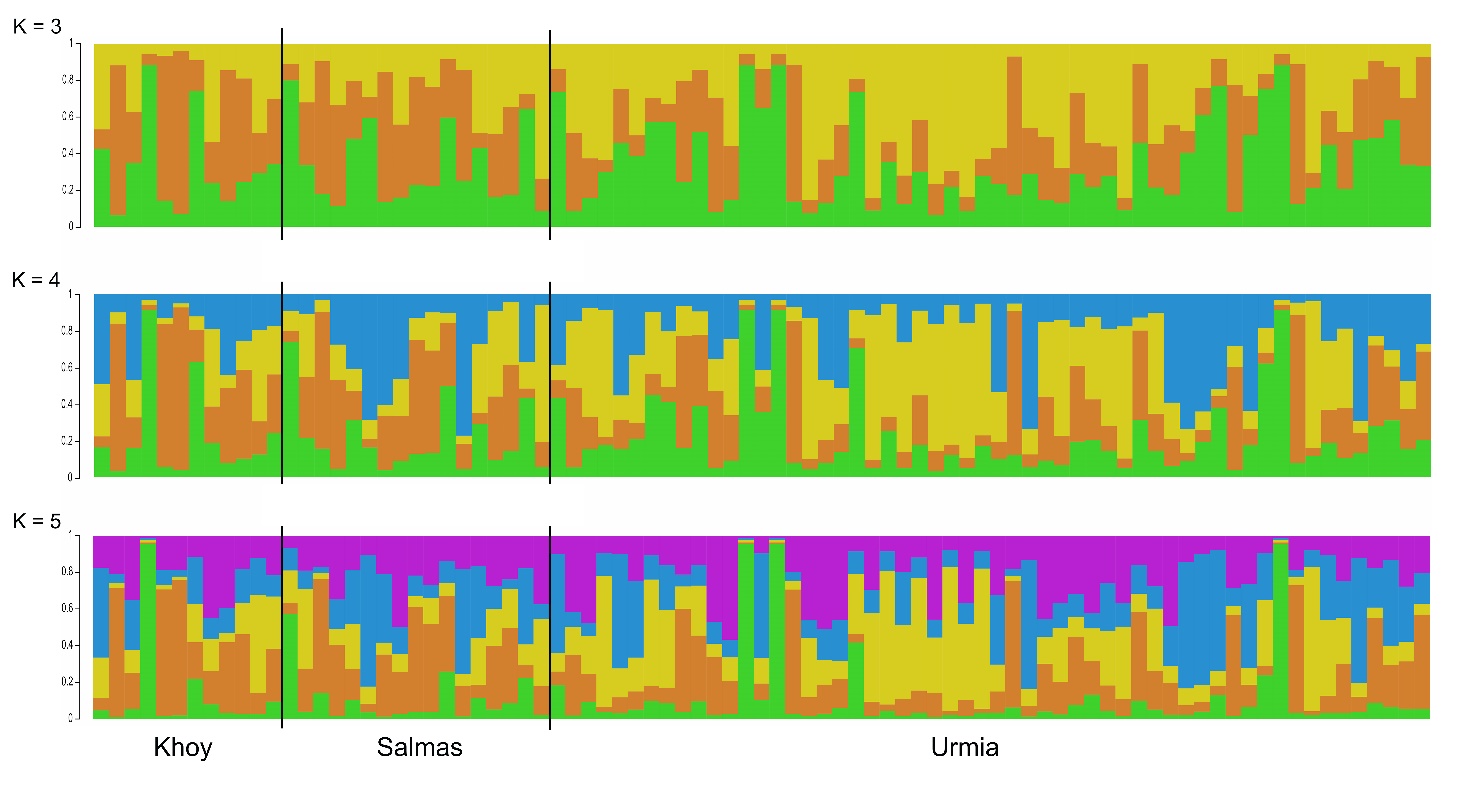


**Supplementary Figure S5.** Assignment of 84 ISSR multilocus genotypes (MLGs) of *Sclerotinia sclerotiorum* from West Azarbaijan province of Iran, into three, four and five genetic clusters (*K*).


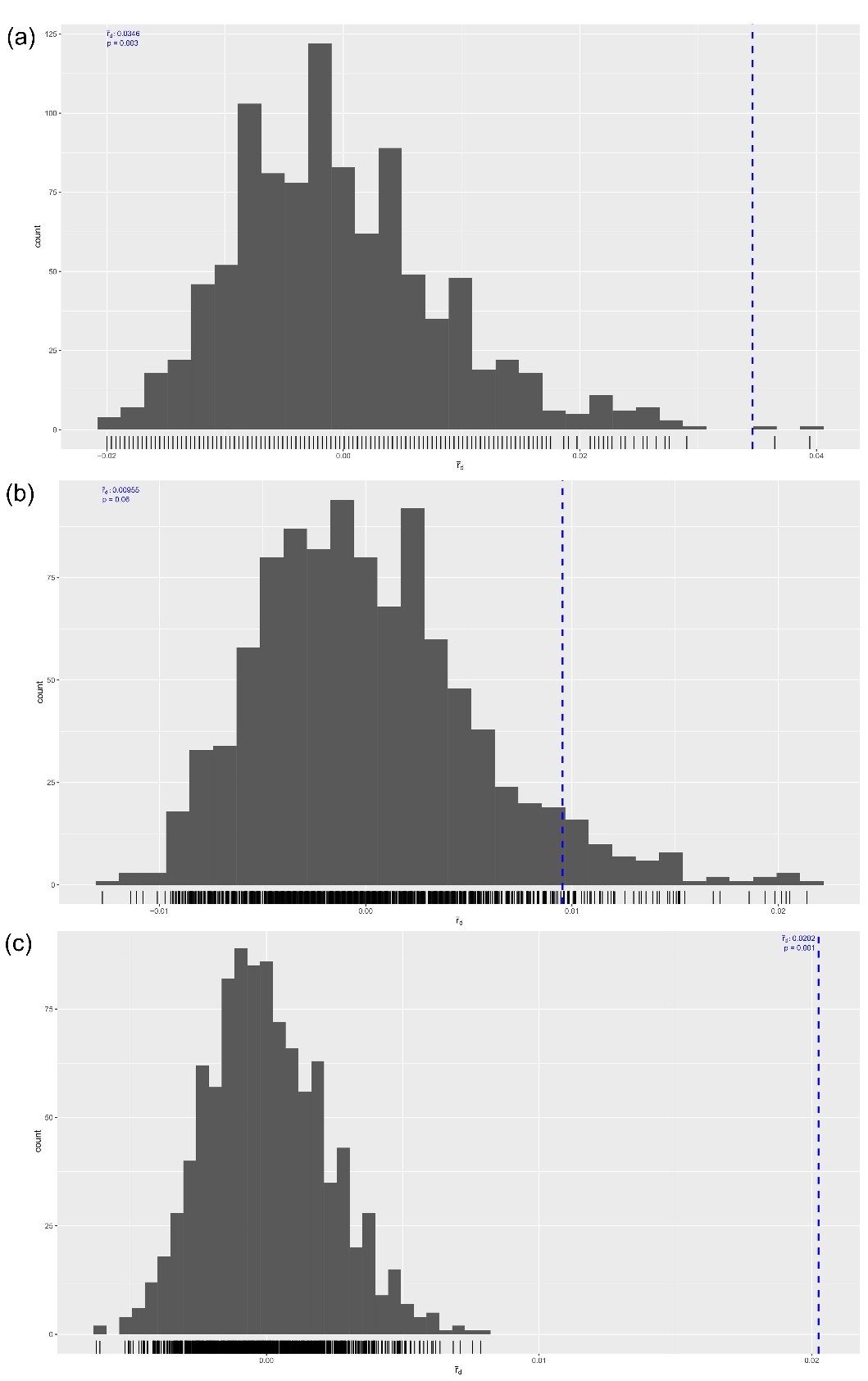


**Supplementary Figure S6.** Estimates of unbiased multilocus linkage disequilibrium index ($\overline{r}$*_d_*) for clone-corrected datasets of *Sclerotinia sclerotiorum* from Khoy (a), Salmas (b) and Urmia (c) populations, calculated using ISSR data in the package *poppr* v. 2.8.7. Deviation of $\overline{r}$*_d_* values from the null hypothesis of linkage equilibrium was assessed by 1000 permutations per population dataset.


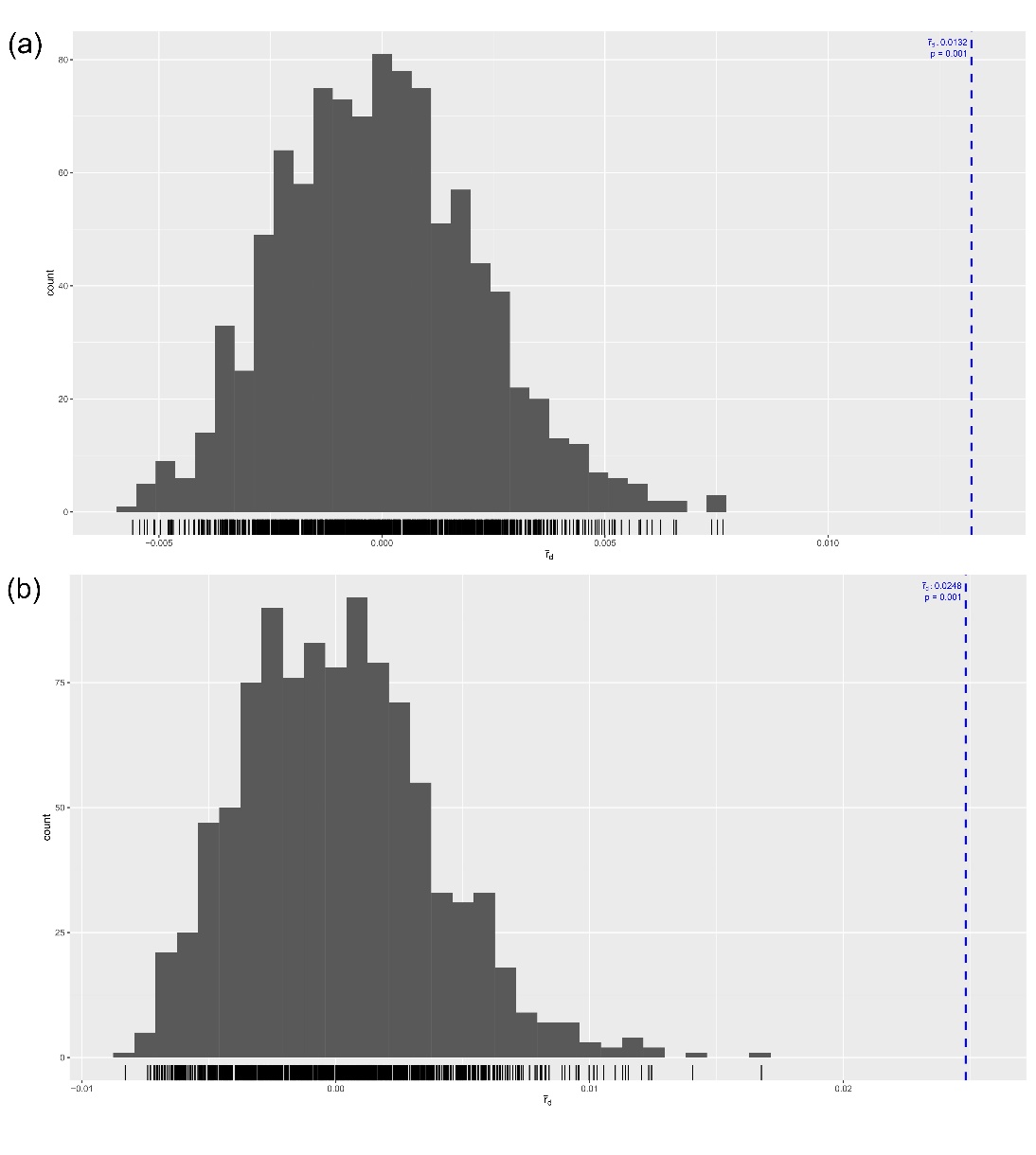


**Supplementary Figure S7.** Estimates of unbiased multilocus linkage disequilibrium index ($\overline{r}$*_d_*) for clone-corrected datasets of *Sclerotinia sclerotiorum* from sunflower (a) and cabbage (b), calculated using ISSR data in the package *poppr* v. 2.8.7. Deviation of $\overline{r}$*_d_* values from the null hypothesis of linkage equilibrium was assessed by 1000 permutations per population dataset.

**
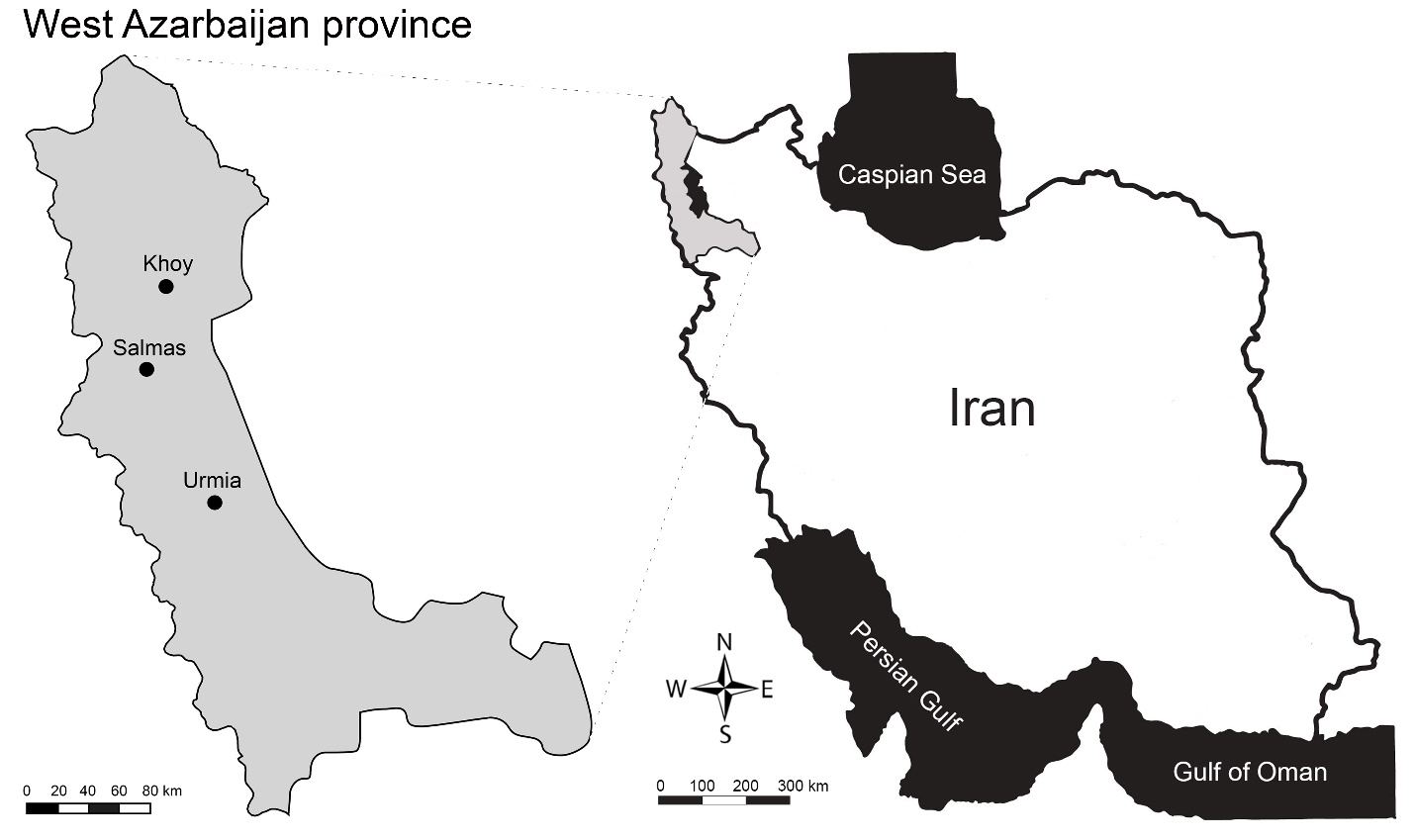
**

**Supplementary Figure S8.** Sampling locations (Urmia, Salmas and Khoy) of *Sclerotinia sclerotiorum* populations in West Azarbaijan province, Iran. The maps created using the Free and Open Source QGIS v. 3.22 (https://qgis.org).
